# Cleavage of SNAP25 and Its Shorter Versions by the Protease Domain of Serotype A Botulinum Neurotoxin

**DOI:** 10.1371/journal.pone.0095188

**Published:** 2014-04-25

**Authors:** Rahman M. Mizanur, Robert G. Stafford, S. Ashraf Ahmed

**Affiliations:** Department of Cell Biology and Biochemistry, Molecular and Translational Sciences Division, United States Army Medical Research Institute of Infectious Diseases, Fort Detrick, Maryland, United States of America; Aligarh Muslim University, India

## Abstract

Various substrates, catalysts, and assay methods are currently used to screen inhibitors for their effect on the proteolytic activity of botulinum neurotoxin. As a result, significant variation exists in the reported results. Recently, we found that one source of variation was the use of various catalysts, and have therefore evaluated its three forms. In this paper, we characterize three substrates under near uniform reaction conditions using the most active catalytic form of the toxin. Bovine serum albumin at varying optimum concentrations stimulated enzymatic activity with all three substrates. Sodium chloride had a stimulating effect on the full length synaptosomal-associated protein of 25 kDa (SNAP25) and its 66-mer substrates but had an inhibitory effect on the 17-mer substrate. We found that under optimum conditions, full length SNAP25 was a better substrate than its shorter 66-mer or 17-mer forms both in terms of *k_cat_*, *K_m_*, and catalytic efficiency *k_cat_*/*K_m_*. Assay times greater than 15 min introduced large variations and significantly reduced the catalytic efficiency. In addition to characterizing the three substrates, our results identify potential sources of variations in previous published results, and underscore the importance of using well-defined reaction components and assay conditions.

## Introduction

The “anthrax letter” scare in the fall of 2001 [1] generated renewed interest in finding remedies to real and perceived bio threat agents. These interests include development of sensitive detection methods for environmental samples as well as dependable activity assay methods to screen large compound databases for potential drug candidates against these bio threat agents. One such bio threat agent is botulinum neurotoxin (BoNT), the most toxic compound known to humans [2]. Its ultimate toxicity relies on a zinc-endopeptidase activity on a neuronal protein. For more than a decade, this protease activity has been the intense focus as a target of inhibitor screening for small molecule drug development. Inhibitory properties of a handful of available inhibitors obtained from these efforts often are not in agreement to each other [3]–[5] because of variations in the particular assay methods employing various catalysts, substrates, and reaction components. An investigation into the effects that these variations have upon enzyme activity is extremely important for adoption of a balanced and appropriate assay conditions in drug discovery [6].

BoNT is a secretory protein produced primarily by several strains of *Clostridium botulinum* [2], [7]. Depending on the bacterial strain producing the BoNT, it has traditionally been classified into seven distinct serotypes, designated as BoNT/A through BoNT/G. Of these, BoNT/A afflicts humans most, followed by BoNT/B and BoNT/E. Although serologically different, all have very similar primary, secondary, tertiary, and quaternary structures. They are produced as single polypeptides of ∼1300 amino acid residues. A posttranslational cleavage, either in the bacterial or host environment cleaves this polypeptide approximately 440 amino acids from the N-terminus resulting in a large C-terminal heavy chain (HC) binding domain, and a small N-terminal light chain (Lc) catalytic domain. Lc is a zinc-endopeptidase in which zinc, bound at the active site, is essential for its enzymatic activity. HC and Lc however remains linked through a conserved disulfide bond. HC is again divided into two subdomains of N-terminal heavy chain (HcN) and C-terminal heavy chain (HcC).

A number of laboratories have synthesized scores of peptides and peptidomimetics [4], [8]–[15], or have synthesized and screened tens of thousands of compounds in small molecule libraries [3], [16]–[22] as inhibitors of BoNT catalytic activity. Most of these efforts however employed a unique combination of BoNT catalyst, its cognate substrate, and a particular assay method. Results from these assays, however, are not always in agreement [3]–[5]. The disagreement of published results poses a major impediment for selecting a useful scaffold that would provide a relevant model for screening small molecules as inhibitors of Lc activity.

Two major sources of discrepancies are (a) the choice of the Lc catalyst and (b) the choice of a substrate (23,24) (Table 1). A third potential source of discrepancy results from the (c) variations in the reaction composition. Other apparent reasons that often remains ignored are (d) reaction time and (e) the analytical technique used to follow the reaction. A recent review has addressed these aspects in more detail [23].

**Table 1:** Substrates, LcA catalytic activity assay methods and results.

Substrate	Assay Method	*K_m_* (µM)	*K_cat_* (Sec^−1^)	Reference
Live mice	Lethality	-	-	[50]
66-mer SNAP25	SDS-PAGE	25	11	[29]
		16	60	[30]
	HPLC	27	<3	[31]
SNAP25	SDS-PAGE	41	2	[51]
GST-SNAP25		14 (64*)	5 (1*)	[52]
GST-SNAP25	SDS-PAGE	10	17	[53]
	HPLC	51* (106*)	10* (4*)	[54]
	ELISA			[55]
SNAP25 (in phrenic hemidiaphraghm)	Electrophysio-logical			[56]
17-mer SNAP25	HPLC	1000	23	[26], [27]
	HPLC	3000–5000	12	[28]
	HPLC	3400	9	[25]
	UPLC	1000	24	[12]
	UPLC	1513	28	This study
SNAPtide™	ALISSA			[57]
SNRTRIDEAN[dnpK]RA[daciaC]RML	Fluorescence	10	7	[26]
(FITC)-T_D_RIDQANQRATK(DABCYL)nL-amide	Fluorescence			[58]
(FITC)-AT_D_RIDQANQRATK(DABCYL)nL-amide	Fluorescence	19		[32]
SNAPtide520	Fluorescence			[33]
SNAPtide521 (FITC/DABCYL)		55		[52]
-SNAPtide522				
CFP-SNAP25_141–206 –_YFP	Fluorescence	0.7	4.1	[59]

*The catalyst used in these cases was whole BoNT/A toxin instead of only LcA in all other cases.

It was not until the availability of comparative data on catalytic constants with and without several types of inhibitors towards four different versions of the catalytic BoNT/A Lc [5], [24], [25] that a clear picture of the reason for discrepancy emerged. Inhibition constant *k_i_* or the extent of inhibition depended on which of the several C-terminally truncated BoNT/A Lc was used [5]. In the past, we have demonstrated that a full length Lc free from rest of the BoNT/A molecule is the most catalytically active species [25]. In light of the inhibitor development problems, we extended that study to include two C-terminally truncated LcA and demonstrated that a full length BoNT/A Lc containing 1–448 residues has the highest catalytic activity because its C-terminal appeared to play a product removal role from the active site of LcA [24]. There was little variation in the substrate *K_m_* catalyzed by these Lcs and by various BoNT/A forms [25].

The cellular target for BoNT/A or its LcA is the 206-residue SNAP25. For convenience, investigators have often used two versions of SNAP25 [12], [25]–[28]: a truncated 66-mer [29]–[31], or a shorter 17-mer version [12], [25]–[28], in addition to a modified Forster resonance energy transfer (FRET) version of the 17mer [3], [32], [33] as the substrate. Data compiled in Table 1 using various forms of the substrate, show that the *K_m_* and *k_cat_* values vary considerably, even if the same substrate is used. This is probably due to major differences in the buffer, reaction component, or the particular analytical tool used. However, except for the 17-mer, and cyan fluorescent protein (CFP) and yellow fluorescent protein (YFP)-tagged CFP-SNAP25_141–206 –_YFP substrates, properties of the rest have not been fully characterized. It is often argued that the 66-mer is a more reasonable counterpart of the full length SNAP25 substrate for LcA. However, no systematic investigation comparing these substrates under a standard set of conditions has been done so far.

Depending upon the concentration, addition of zinc and dithiothreitol (DTT) to the LcA reaction mixtures can be both stimulating and inhibitory [34]. Similarly, both *K_m_* and *k_cat_* of the 17-mer substrate are dramatically affected by increasing concentrations of bovine serum albumin [35]. Salts and buffer components like NaCl, Na-phosphate, tris.HCl, and ethylenediaminetetraacetate (EDTA) are inhibitory to BoNT/A activity [34], [36]. Presence of these components in the LcA or substrate preparations or in the reaction mixtures can potentially give misleading activities and false inhibitory results. Thus, it is very important that activity of one standard LcA catalyst be determined using several of the currently used substrates, so that the effects of various additives on the rate of the reaction can be evaluated. Results obtained from such a study will allow a direct comparison of the properties of LcA and the substrates for a more realistic evaluation of inhibitor screening.

In this backdrop, the current investigation compares the substrate properties of the 17-mer, the 66-mer, and the full length SNAP25 with the most active BoNT/A catalyst under near identical assay conditions. We also examined the effects of several additives that have been in use in each of these assays. Our results provide a direct comparison of these effects demonstrating for the first time that reaction components, particularly NaCl, exert completely different effects depending upon which substrate is used. Additionally, we show that the reaction time has a profound effect on the enzyme constants, and the full length SNAP25 is by far the best substrate that yields the lowest *K_m_* and highest *k_cat_* values.

## Results

### 17-Mer substrate

Previously, we reported that a LcA preparation solubilized from inclusion bodies behaved very similar to that of whole BoNT/A toxin when assayed with the synthetic 17-mer substrate [34]. These similarities included activity stimulation by BSA [27], and in *K_m_* and *k_ca_*_t_ values [34], [35]. We had also reported [34] that the solubilized LcA activity was inhibited by including 5 mM DTT that could be neutralized by the addition of ZnCl_2_. Conversely, in the absence of DTT or β-mercaptoethanol, the reaction was inhibited by increasing concentrations of ZnCl_2_. We had concluded that our LcA preparation, having stoichiometric amounts of zinc, behaved similar to other Zn-metalloproteases [37]. We repeated these experiments with the new LcA preparation purified from soluble extracts and found essentially same behavior was displayed by adding BSA, ZnCl_2_, and DTT (data not shown). Therefore, in routine assays we included 0.2 mg/ml BSA but omitted ZnCl_2_ and DTT. Under these conditions and employing a 17-mer substrate concentration of 0.13, 0.27, 0.33, 0.44,0.67, and 1.17 mM, we calculated its *K_m_* as 1.5 mM and a *V_max_* of 33.6 mM/min/mg (*k_cat_* of 28/sec) (Table 1–3). In terms of *V_max_* using the 17-mer substrate, this LcA preparation has the highest activity reported in the literature [28], [34], [35].

**Table 2:** Dependence of *K_m_*, *k_cat_* and *k_cat_/K_m_* for full length SNAP25 substrate on the LcA reaction time.

Time (min)	*K_m_* (µM)	*k_cat_* (Sec^−1^)	*k_cat_*/*K_m_* (µM/Sec)
5	29.8±4.02	73.9±5.32	2.47
10	31.6±4.35	76.8±5.63	2.43
15	38.5±1.19	78.2±1.38	2.03
30	67.4±1.61	88.4±1.14	1.31
45	180±18.8	159±14.9	0.883
60	189±14.6	134±8.75	0.709

The values were calculated by hyperbolic curve fitting by Michaelis-Menten equation of the data presented in Figure 5B.

**Table 3:** Steady state kinetic constants for LcA reactions utilizing various SNAP25 substrates.

SNAP25 Substrate	*K_m_* (µM)^a^	*k_cat_* (Sec^−1^)^a^	*k_cat_*/*K_m_* (µM/Sec)
17-mer^b^	1513±281.8	28±3.5	0.019
66-mer	32.43±0.9281	23±0.22	0.72
Full Length	33.34±4.592	76±2.2	2.3

a The values are averages of results from 5, 10, and 15-min reactions reported in Table 2.

b 10 min incubation data. Kinetic parameters were obtained by hyperbolic Michaelis-Menten curve fitting (R^2^ = 0.99345) of experimental 5 replicate data.

**Figure 1:**
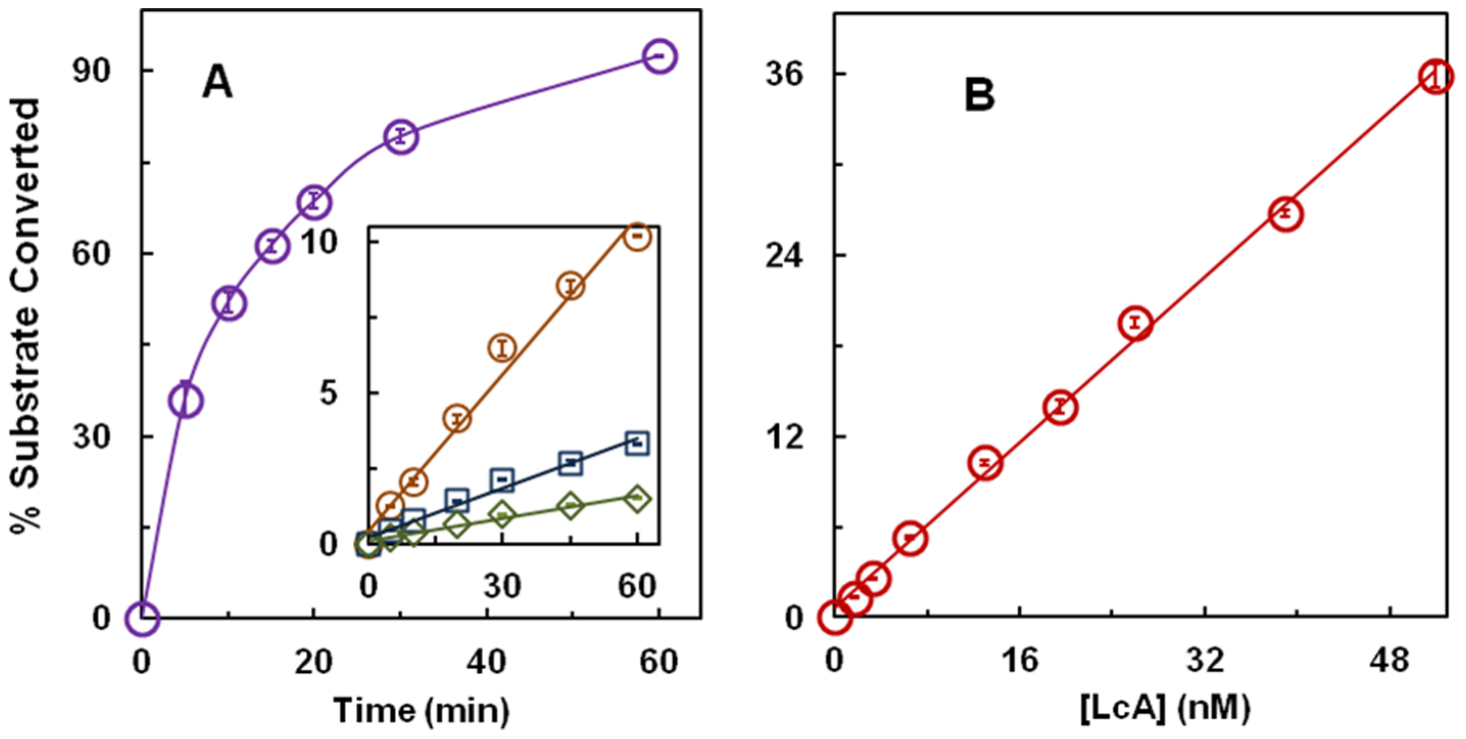
**A**, Time course of reaction of LcA utilizing the 17-mer peptide (0.5 mM) as a substrate in the presence of 0.2 mg/ml BSA in 50 mM NA-HEPES, pH 7.4 at 37°C. LcA concentrations used was 51 nM (purple circle), and in the inset, 1.6 nM (gold circle), 0.8 nM (blue square), and 0.4 nM (green diamond). Each data point is an average of 5 assays. **B**, 5-Min data for each of the LcA concentrations used in A and others not shown are plotted as a function of LcA concentration. Bars in both panels represent standard deviations. These results show that (a) at high enough LcA concentration, the time course becomes nonlinear (due to substrate depletion), (b) LcA concentration as low as 0.4 nM remains stable (in the presence of 0.2 mg/ml BSA) at 37°C for at least 1 hour, and (c) if enough substrate is available, the reaction rate is linear to LcA concentration between 0.4 and 51 nM.

Because LcA is now routinely used to screen for BoNT/A protease inhibitors using a variety of reactions such as in high throughput formats, the purity, concentration, time of incubation, and its temperature dependent stability needs to be carefully established. LcA used in this study is a highly pure protein showing only one stained band in sodium dodecyl sulfate-polyacrylamide gel electrophoresis (SDS-PAGE) and western blot [38]. Figure 1A shows that 51 nM LcA rapidly consumes the 17-mer substrate and that substrate conversion-time linearity is clearly lost after 5 min, the first time point at which the reaction measurement can be recorded accurately. By lowering the LcA concentration to 0.4–1.6 nM, linearity was observed for 60 min although detection of product at earlier time points, particularly with 0.8 and 0.4 nM LcA yielded results with high standard deviation. Yet the results in Figure 1A inset indicate that in the presence of 0.2 mg/ml BSA, LcA at concentration as low as 0.4 nM remains stable at 37°C for at least 1 hour. By plotting the amount of substrate converted (or product formed) in 5 min as a function of LcA concentration, we found a fairly linear relationship between LcA concentration and extent of substrate conversion up to 36%. In addition to characterizing the LcA preparation as a stable protein under the specified conditions, results in these two figures allow us to select a combination of LcA concentration, reaction time, and sensitivity of detection that is most suitable for a particular assay including high throughput screening platforms using this substrate.

### Behavior of LcA towards the full length SNAP25 and its synthetic 66-mer peptide substrate

The commercially-obtained full length SNAP25 is a reasonably pure protein of 206 residues. The protein concentration was verified by complete digestion with LcA (data not shown). SNAP25 is supplied by the vendor in 25 mM tris.HCl buffer containing 2 mM EDTA. We [34] and others [39] have demonstrated that tris.HCl is inhibitory to BoNT activity and that EDTA, in addition to being a metal chelator, is also an inhibitor of LcA activity. Therefore the full length SNAP25 was extensively dialyzed against 50 mM Na-HEPES, pH 7.4 before using in the assays.

**Figure 2:**
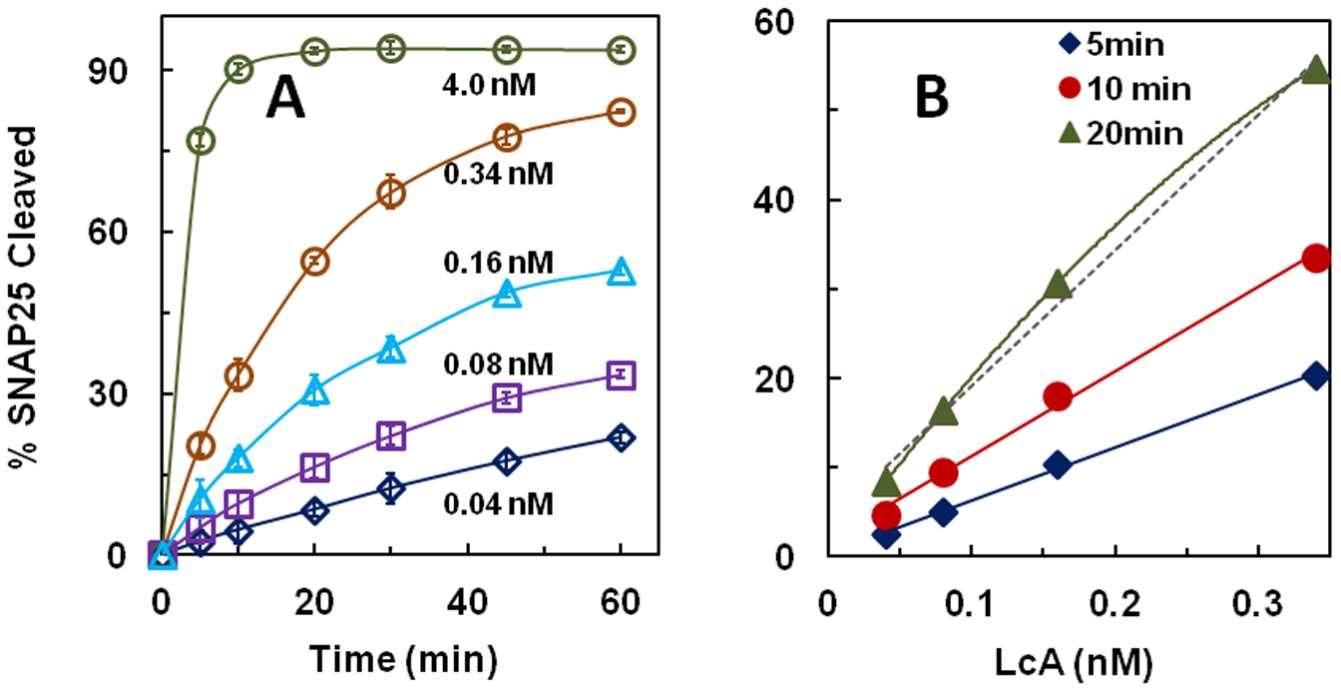
**A**, Time course of reaction of LcA utilizing the full length SNAP25 (11.3 µM) as a substrate in the presence of 0.2 mg/ml BSA, 0.25 mM ZnCl_2_ and 5 mM DTT in 50 mM NA-HEPES, pH 7.4 at 37°C. LcA concentrations ranging from 0.04 nM to 4.0 nM were as indicated. Each data point is an average of 5 assays. Bars represent standard deviations. **B**, 5, 10 and 20-min data for four of the LcA concentrations used in **A** are plotted as a function of LcA concentration. The dotted line is the best fit (y = mx + c) of the 20-min data points while the curved lines connect the actual data points for each of 5-min, 10-min and 20-min data points. These results show that (a) above 0.04 nM LcA concentration, the time course becomes nonlinear (due to substrate depletion), (b) LcA concentration as low as 0.04 nM remains stable at 37°C for at least 1 hour, and (c) if enough substrate is available, the reaction rate should be linear between 0.04 and 4.0 nM LcA concentration.

Incubation of various LcA concentrations with a fixed, 11.3 µM concentration of SNAP25 yielded results as depicted in Figure 2. At the highest LcA concentration, more than 90% of the substrate was converted into products while at the lowest LcA concentration, only ∼20% substrate was consumed in 60 min. Please note that the lowest concentration (0.04 nM) of LcA used in this experiment is an order of magnitude lower than the lowest concentration used in previous experiments (Figure 1), because the substrate SNAP25 concentration was 45-fold lower than that used with the 17-mer peptide substrate due to almost 50-fold lower *K_m_* (see later). Even at this low concentration of 0.04 nM LcA, incubation at 37°C for 1 hour did not denature the enzyme, as was evidenced by the fact that time-dependent product formation maintained a linear relationship during the incubation (Figure 2A). The major difference observed in these experiments versus results shown in Figure 1A was that the SNAP25 substrate did not have a time dependent linearity with LcA concentration above 0.08 nM having incubations longer than 10 min (Figure 2B). This loss of linearity of the reaction must be due to depletion of substrate because the lowest LcA concentration (0.04 nM) yielded a straight line for 60 min (Figure 2A). Although a linear relationship between the % product formed in 5 and 10 min at 0.04–0.4 nM LcA concentrations (Figure 2B) was obtained, there was a nonlinear tendency at the 20-min incubations that was due to substrate depletion as noted before. Implications of these results are more thoroughly addressed later in Table 2.

These results underscore the importance of choosing the right LcA concentration, and the time of enzymatic reaction in devising a standard assay protocol. The LcA concentration-dependent reaction linearity shown in Figures 1B and 2B also show that the LcA preparation does not contain contaminants that would complicate the steady-state kinetics and data described below.

**Figure 3:**
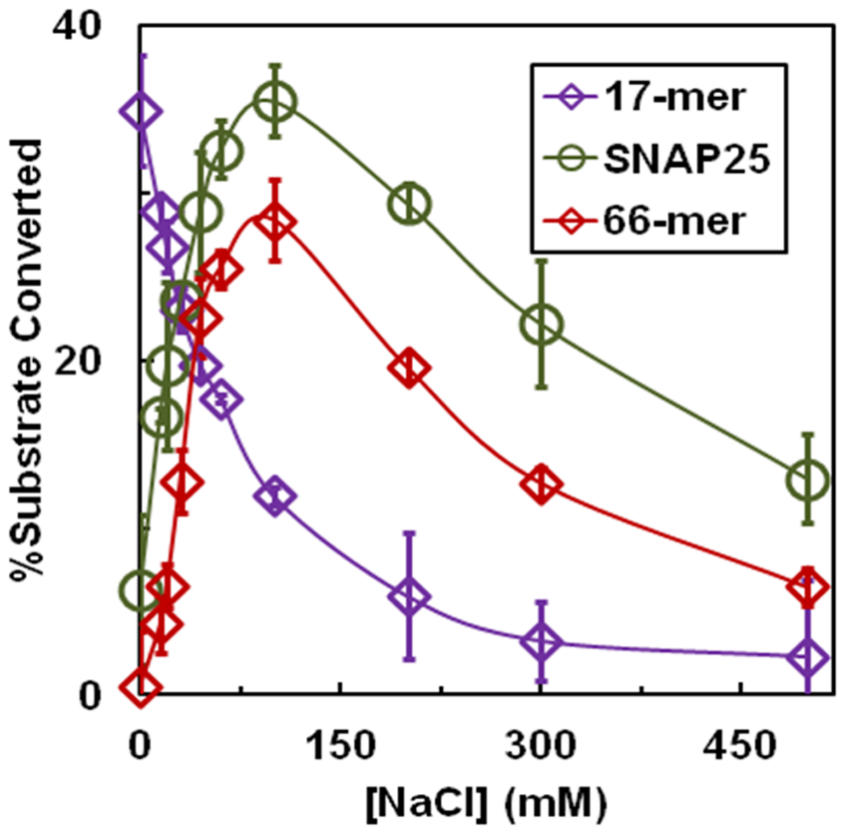
Substrate-dependent effects of sodium chloride on the catalytic activity of LcA. The 30-µl reaction mixtures containing 0.2 mg/ml BSA, 12 µM SNAP25 or 66-mer substrate +1.0 nM LcA, or 0.5 mM 17-mer substrate +50 nM LcA in 50 mM Na-HEPES, pH 7.4. The choice of 50-fold difference in LcA concentration was based on the fact that the SNAP25 substrate concentration used was 42-fold lower than the 17-mer substrate because of a ∼45-fold difference in the *K_m_* of SNAP25 or its 66-mer with the 17-mer (see later in Table 3). The SNAP25 and 66-mer substrate reaction mixtures also contained 5 mM DTT, and 250 µM ZnCl_2_, and the reactions were allowed to proceed for 10 min, while the 17-mer reaction was allowed to proceed for 5 min. Addition of 5 mM DTT and 250 µM ZnCl_2_ to the 17-mer substrate reactions yielded results identical to those without them as shown in this figure.

Data presented in Figure 3 show that increasing the NaCl concentration to 100 mM in the reaction mixture containing SNAP25 and LcA, dramatically increased LcA activity more than sevenfold. Higher concentrations of NaCl caused a decrease in LcA activity. The same was also observed when the 66-mer peptide was used as a substrate. These stimulating effects of NaCl towards the full length and 66-mer versions of SNAP25 are in quite contrast to its inhibitory effect towards the 17-mer substrate as shown in Figure 3 and previously noted elsewhere [34]. The most logical explanation for the stimulating effect of NaCl is that it disrupts nonspecific, inter- and intra-molecular protein-protein interactions between the large molecules of SNAP25, the 66-mer, and LcA. Such interactions will limit the availability of monomeric forms of the molecules needed to act as substrates. The 17-mer peptide being much smaller than the full length SNAP25, is most likely free from such interactions.

### Effects of DTT, BSA, and Zn on LcA activity with SNAP25 and the 66-mer peptide

**Figure 4:**
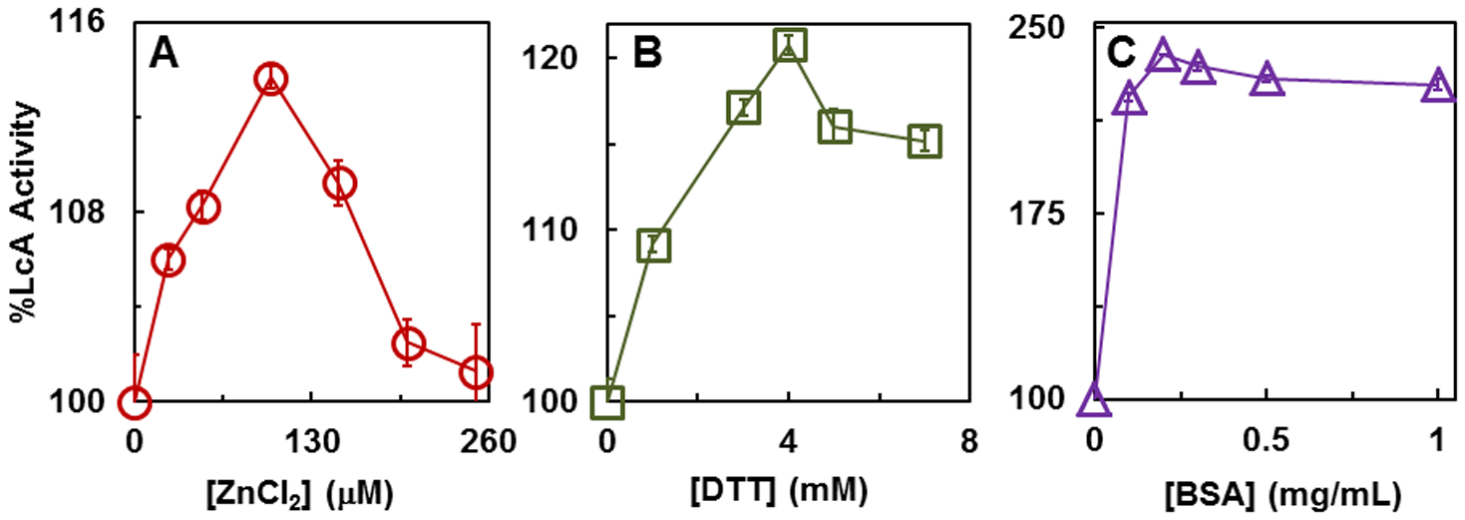
Effects of ZnCl_2_ (A), DTT (B), and BSA (C) on the catalytic activity of LcA using SNAP25 as a substrate. The 30-µl reaction mixtures containing 12 µM SNAP25, 0.34 nM LcA in 50 mM Na-HEPES, pH 7.4 were incubated at 37°C for 10 min. In addition, the reaction mixtures contained 5 mM DTT in **A**, 250 µM ZnCl_2_ in **B**, and 5 mM DTT and 250 µM ZnCl_2_ in **C**.

**Figure 5:**
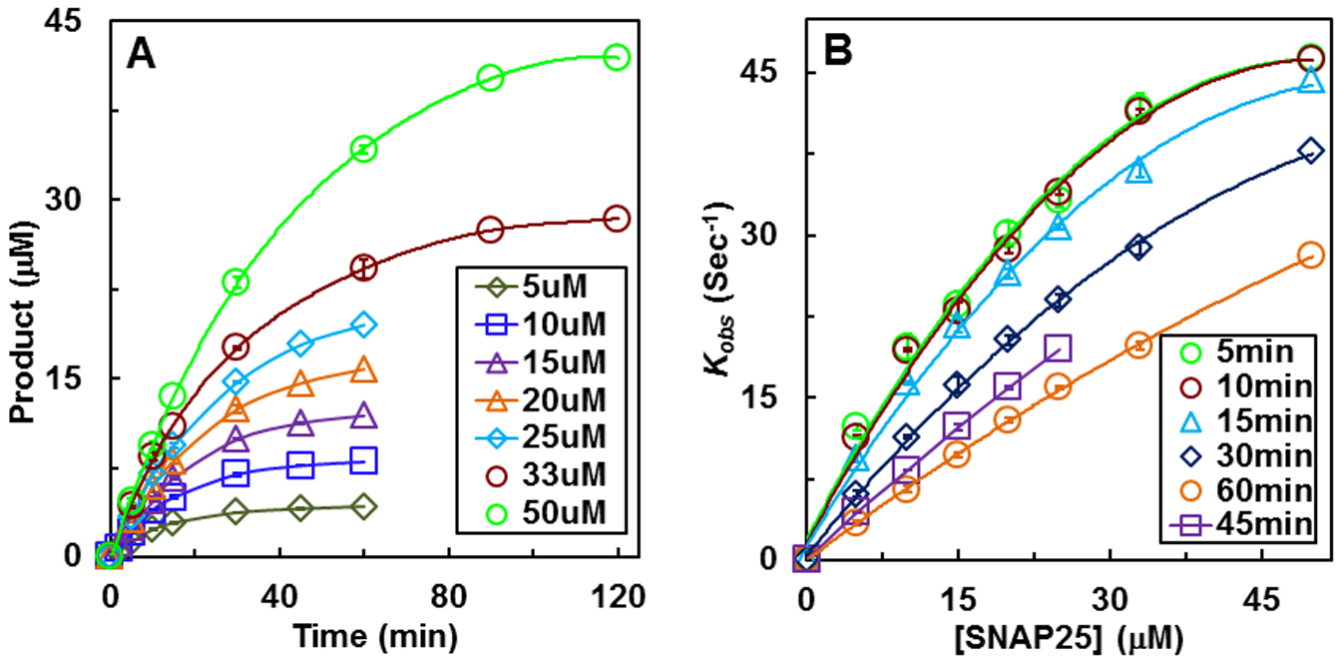
Time course of LcA reaction using various concentrations of SNAP25 (A), and linear plots of reaction rate (*k_obs_*) calculated at various time points versus SNAP25 concentrations (B). Each reaction mixture contained 0.2/ml BSA, 5 mM DTT, and 250 µM ZnCl_2_, 0.34 nM LcA, and 50 mM Na-HEPES, pH 7.4. The bars inside symbols represent standard deviation of three assays. The lines connecting the data points in B were generated by curve fitting using the Michaelis-Menten equation *k_obs_* = (*k_cat_*×[S])/(*K_m_*+[S]) in a KaleidaGraph software package. *K_m_ and k_cat_* values derived from these curves are reported in Table 2.

Unlike the whole BoNT/A toxin which contains an inter chain disulfide bond, LcA does not contain a disulfide bond. However SNAP25 containing four cysteine residues located near the middle of the molecule, have a propensity to form mixed disulfide bonds [40], [41] and other oxidation products that lead to insolubility and precipitation. The same would be expected with the 66-mer peptide because it contains the same four cysteine residues. Therefore 5 mM DTT was included in all LcA reactions using these two substrates. Reaction mixtures having no additive such as DTT, ZnCl2, BSA, or the 57-mer peptide were treated as controls in the respective experiments. DTT has a propensity to form complexes with divalent metal ions [42] such as the Zn^++^ bound at the LcA active site and could potentially inhibit LcA activity. Therefore, we measured LcA activity in the presence of 5 mM DTT and increasing concentrations of ZnCl_2_. There was a slight stimulation of the activity up to 100 µM ZnCl_2_ after which the activity declined with increasing concentrations although at 250 µM the activity remained above the control 100% (Figure 4A). Similarly, at a fixed 250 µM concentration of ZnCl_2_, slight stimulation of LcA activity was observed when 1–8 mM DTT was added to the reaction mixture (Figure 4B). Although optimal activity was obtained at 0.1 mM ZnCl_2_ and 4 mM DTT, there was little difference with the activities at 0.25 mM ZnCl_2_ (Figure 4A) and 5 mM DTT (Figure 4B), the optimal concentrations reported [34] and used earlier [4], [12], [14], [24], [26], [27], [34], [43]–[45].

The stimulating effect of BSA on LcA activity was more evident than either DTT or ZnCl_2_ as the activity was more than doubled by the addition of 0.1 mg/ml BSA (Figure 4C). This level of stimulation did not change with up to 1 mg/ml BSA. We demonstrated earlier that LcA is prone to precipitation but BSA provided a stabilizing effect by keeping it more soluble [46]. Saturation of SNAP25 cleavage activity stimulation by the addition of low BSA concentrations (0.1∼0.2 mg/ml) (Figure 4C) compared to ∼2 mg/ml BSA needed for optimum cleavage of the17-mer substrate [27], may be related to the fact that the large SNAP25 substrate itself provides the stabilizing effect on LcA.

Results obtained in these experiments led us to formulate a standard reaction mixture recipe that contains 0.2 mg/ml BSA, 5 mM DTT and 0.25 mM ZnCl_2_ to use with SNAP25 or its 66-mer as substrates. In routine assays, the latter concentrations are more convenient to prepare from concentrated stocks.

### Steady state kinetic constants using the three different substrates

The Michaelis constant *K_m_* for various forms of the 66-mer or the full length SNAP25 has been reported to range from 14 µM to 64 µM by various laboratories (Table 1), reflecting a fourfold to fivefold difference. A much more pronounced difference in the values of *k_cat_* (2–60/sec) using these substrates were also reported. Additionally, substrate differences, source and/or quality of LcA, time of the enzymatic reaction (5 min to 2 hours), and the analytic methods used for quantification varied as well. Another potential reason for the differences in the kinetic constants in Table 1 may be due to different methods of computation used. It occurred to us that because sub-saturating concentrations of the substrate (maximum fivefold above *K_m_*) was used in all cases in Table 1, and the reaction incubation time differed, time-dependent rapid depletion of substrate could account for the large variations in the reported values of *K_m_* and *k_cat_*. We therefore followed the LcA reaction from five to 60 minutes using full length SNAP25, and the 66-mer SNAP25 peptide substrates. Figure 5A shows a representative time course experiment using seven different concentrations ranging from 5 µM to 50 µM of the full length SNAP25. Longer the time of incubation, more products are formed (less substrate is remaining) such that most of the substrate was consumed over 60 min. As a result, except for the 10 min incubation, the *k_obs_* progressively decreased when calculated from the increasing incubation time data, as depicted in Figure 5B. These observations are reflected in the derived values of *k_cat_* and *K_m_* that increased with time of incubation (Table 2). Although both parameters increased with time of reaction, the catalytic efficiency, calculated as *k_cat_*/*K_m_* progressively decreased almost fourfold from 10 min to 60 min incubation (Table 2).

For routine use SNAP25 stock solution can be obtained at a concentration of no more than 40 µM. So there is a practical limit of its concentration in the assays. With this limitation of using low concentrations of the substrate in routine assays, a 5–15 min reaction incubation time appears to provide a reasonably accurate *K_m_* (33.34±4.592 µM) and *k_cat_* (76±2.2/sec) values for full length SNAP25 (Table 3). Essentially identical experiments using the 66-mer as a substrate yielded an almost identical *K_m_* of 32.43±0.9281 µM but almost threefold reduced *k_cat_* of 23±0.22/sec (Table 3). Thus, the 66-mer peptide appears to be a poor substrate when compared with the full length SNAP25. At the same time, the 66-mer substrate with a very similar *k_cat_* but almost 50-fold lower *K_m_* is a better substrate than the 17-mer peptide (Table 3). In terms of *K_m_*, and *k_cat_*/*K_m_*, the 17-mer peptide is the poorest of all the three substrates followed by the 66-mer substrate The full length SNAP25 having the highest *k_cat_*/*K_m_* value, appeared to be the best substrate. Nonetheless, *k_cat_* with the 17-mer is comparable to that with the full-length and the 66-mer substrates as opposed to its very high *K_m_*. Because most of the active and catalytic site interactions of LcA is contained in this 17-mer substrate [15], its low cost and high solubility [27] should make the 17-mer a preferred choice in routine screening for inhibitors of LcA catalytic activity.

### Inhibition of LcA activity by a product of its reaction with substrate

**Figure 6:**
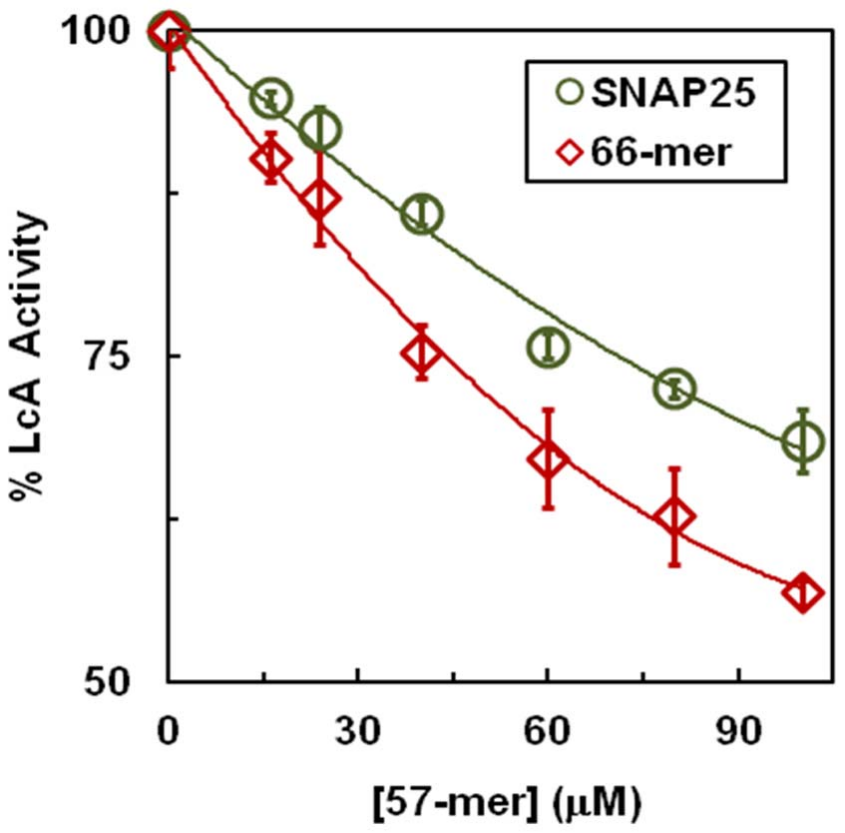
Inhibition of LcA activity by the 57-mer N-terminal product of the enzymatic reaction of LcA on SNAP25 and a 66-mer peptide substrate. Reaction mixtures (30 µl) containing 10 µM substrate, 1.01 nM LcA, 0.2 mg/ml BSA, 5 mM DTT, 0.25 µM ZnCl_2_ and the indicated concentrations of the 57-mer product peptide in 50 mM Na-HEPES, pH 7.4 were incubated at 37°C for 5 min.

Cleavage of the 66-mer substrate with LcA results in a 57-mer N-terminal product and a 9-mer C-terminal product. To investigate if the nonlinearity of the time course of reaction observed in Figure 5A could be partly due to inhibition of the reaction by the formed product, we incubated LcA with SNAP25 in the presence of 50 µM 9-residue C-terminal product or 50 µM 57-residue N-terminal product. Only the 57-mer product affected the LcA activity. Figure 6 shows that irrespective of the two large substrates used, activity of LcA is inhibited by the 57-mer product. The inhibition appears to be more pronounced with the 66-mer substrate than with the full length SNAP25 substrate. In contrast to the inhibition by the 57-mer product, the reaction of LcA with the 17-mer substrate was not inhibited by either its N-terminal 11-mer or the C-terminal 6-mer or peptide products [24]. Only 5–10% inhibition of these larger substrate cleavage reactions at 10 µM peptide (Figure 6) suggest that the nonlinearity of the time course in Figure 5A may not be due to inhibition by the formed N-terminal product. However, we cannot rule out the possibility that the off rate of the formed product might be slower than the on rate of our extraneously added 57-mer product. Recently, we provided evidence for a LcA C-terminus-mediated N-terminal product removal step in the LcA catalysis of the 17-mer substrate reaction [24]. Our unpublished results indicate this might be true with the full length and 66-mer SNAP25 substrate too. In that respect, the decreased *k_obs_* values with increasing substrate concentration observed in Figure 5B may be partly due to inhibition of the reaction by the 57-mer product.

## Discussion

To identify inhibitors of BoNT endoproteolytic activity, several quantitative methods and reagents are used by various investigators [12], [25]–[31]. As a result, there is significant variation in the data obtained which creates confusion in the interpretation of the results [3]–[5]. In many cases, the substrates and reaction components have not been characterized using uniform assay conditions and analytical methods. With the aim to fill this gap, the present investigation compared the behavior of three BoNT/A substrates and several additives that are commonly used in activity measurement assays. We have shown that sodium chloride, a common additive in many enzyme reactions, differently affects the rates of reactions using alternate substrates. We have also shown that incubation time and substrate concentrations affect the calculated values of the reaction rate (*k_obs_*). Based on these experimental demonstrations, we have formulated an assay protocol for each of the 3 substrates (Table 4). Because these substrates approximate the natural SNAP25 substrates more than the modified, FRET substrates, the assay formulations described below should provide a standard reference when comparing activity results using various substrates and methods.

**Table 4:** Standardized recipe for enzymatic activity assays of LcA using three different substrates.

Reaction Component	Substrate		
	17-mer	66-mer	Full length
LcA	25 nM	0.3 nM	0.3 nM
BSA	0.2 mg/ml	0.2 mg/ml	0.2 mg/ml
DTT	-	5 mM	5 mM
ZnCl_2_	-	250 µM	250 µM
NaCl	-	100 mM	100 mM
Na-HEPES	50 mM	50 mM	50 mM
pH	7.4	7.4	7.4
Substrate	0.5 mM	10 µM	10 µM
Incubation temp	37°C	37°C	37°C
Incubation time	5 min	5–15 min	5–15 min

Because both the substrate and enzyme in BoNT assays are proteins, their purification buffers often contain EDTA, NaCl, and phosphate, all of which inhibits BoNT activities [34], [36], [47]. In devising a standard assay protocol, one needs to avoid the presence of these or any known inhibitory buffer or additive in the reaction mixture. If an initial experiment shows substoichiometric zinc bound to the BoNT or its Lc preparation, low concentrations (not to exceed 250 µM) of zinc chloride should be added to the reaction mixture. Including 5 mM DTT helps to overcome the inhibitory effect of zinc [34]. Inclusion of zinc and DTT is more important when the catalyst is whole BoNT toxin or when the substrate is SNAP25 or its 66-mer, all of which contain one or multiple cysteine residues. BSA should be added to protect LcA at low concentration from denaturation before or during the reaction time. NaCl should not be used with the 17-mer or similar substrates, but should be included with full-length or the 66-mer SNAP25 substrate.

Usually the multi-well plate incubation chambers are set at temperatures lower than 37°C. Simultaneous reactions of large number of samples in plates with 24-, 96- or higher number wells will also require longer times of preparation. Therefore in adapting these reactions in multi-well plates, temperature and incubation times may need to be modified in order to provide a robust assay. Lower incubation temperatures will require higher LcA concentrations, but longer incubation times will require lower LcA concentration. For example, we observed that a 30 min incubation at 22°C was required to convert 40% of the substrate into products using 25 nM LcA with 0.5 mM 17-mer substrate or 0.34 nM LcA with 11 µM full length SNAP25 substrate. Similarly, it took 60 min under the same conditions for 60–65% substrate conversions. For comparison, almost all of the substrates were converted into products in 30 min at 37°C.

Although highly desirable, in routine assays it is not possible to use saturating concentration of full length soluble NSF attachment protein receptor (SNARE) or their peptide, or FRET substrates because of (a) solubility, (b) availability, (c) detection limitation of the instrument, and (d) cost. Therefore a compromised highest concentration of substrate near its *K_m_* value (Table 4) should be chosen.

Formulation of the three recipe in Table 4 is based on the consideration that no more than 25% of the substrate will be converted into products. The experiments described in this paper added 5 µl LcA to 25 µl substrate-additive master mix (as described in Table 4) to initiate the reaction. Identical results can also be obtained by adding 5 µl substrate to 25 µl LcA-additive master mix to initiate the reaction. Screening of inhibitors can be accomplished by adding such compounds to the master mix prior to addition of LcA. However, inhibitor libraries may contain compounds that are slow binders of an enzyme. In such a situation, a preincubation of the inhibitor candidates with LcA in the master mix followed by addition of substrate may be desirable and more convenient than adding LcA at the end. In either case, a short vortex for 1 sec immediately after the final addition must be done to ensure complete mixing of this small 30 µl volume.

In our standard practice, 25 µl master mix in a 1.8 ml screw capped eppendorf tube is preincubated for 5 min at 37°C. 5 µl LcA or substrate at ambient temperature is added to the eppendorf tube and immediately vortexed followed by incubation of the capped tube at 37°C. The reaction is stopped by acidifying the mixture with 90 µl of 1% TFA. A brief spin at 12,000 g for 2 min helps to precipitate any formed particulate material ensuring better chromatographic column performance. Fifty to 110 µl of the supernatant is transferred into UPLC™ or HPLC vials for product analyses.

## Materials and Methods

### Materials

Recombinant BoNT light chain of serotype A (LcA) was purified as described [38], [48], [49]. Human SNAP25 sequence-derived substrates and products were as follows: 66-mer substrate peptide (141-ARENEMDENLEQVSGIIGNLRHMALDMGNEIDTQNRQIDRIMEKADSNKTRIDEANQRATKMLGSG-206), 57-mer product peptide (141-ARENEMDENLEQVSGIIGNLRHMALDMGNEIDTQNRQIDRIMEKADSNKTRIDEANQ-197), 17-mer substrate peptide (SNKTRIDEANQ-RATKML), 11-mer product peptide (SNKTRIDEANQ), 9-mer product peptide: (198-RATKMLGSG-206), and the 6-mer product peptide (RATKML). All substrate peptides having N-terminal acetylated and C-terminal amidated, were custom-synthesized and purified to >95% by Peptide2.0, (Chantilly, VA 20153). The products of LcA reaction on the 17-mer, N-acetylated SNKTRIDEANQ (not C-amidated) and C-amidated RATKML (not N-acetylated) were also from the same vendor.

Full length recombinant human SNAP25 (1 mg/ml or ∼40 µM), purchased from GeneWay Biotech Inc. (Santa Clara, CA) was extensively dialyzed against 50 mM HEPES pH 7.4, and saved as small aliquots at −20°C until use. If a higher concentration of the protein was required, it was concentrated on a Centricon-10 (Amicon) ultrafiltration unit.

### Enzymatic activity assays

Concentration of LcA was determined from its *A^1%^* ( = 10) at 280 nm in an Agilent 8453 diode array spectrophotometer [34]. Activity assays were based on ultra performance liquid chromatography (UPLC™) or high performance liquid chromatography (HPLC) separation and measurement of the cleaved products from the SNAP25 substrate. A master reaction mixture lacking the LcA was prepared and its aliquots were stored at −20°C. Stocks of 0.05–0.07 mg/ml LcA in 50 mM Na-HEPES, pH 7.4 containing 0.05% Tween-20 were also stored at −20°C. Before assay, a Lc stock was thawed and diluted further in 50 mM NA-HEPES, pH 7.4, containing bovine serum albumin (BSA). At the time of assay, 5 µl of diluted LcA was added to 25 µl of the thawed master mix to initiate the enzymatic reaction. Components and final concentration in this 30-µl reaction mixture were 0.005–1.5 mM substrate, 0.2 mg/ml BSA, 0.4–50 nM (or 0.04–4 nM as indicated) LcA, 0.25 mM ZnCl_2_, 5 mM dithiothreitol (DTT), and 50 mM *N*-2-ydroxyethylpiperazine-*N*-2-ethanesulfonic acid whose pH was adjusted with NaOH (Na-HEPES) pH 7.4. Because DTT and ZnCl_2_ had no effect on the kinetic constants [43] or specific activity [12] of LcA employed in this study using the 17-mer substrate, these reagents were omitted from the 17-mer substrate reactions. After 5 or 10 min (depending on the particular experiment) incubation (37°C), reactions were stopped by adding 90 µl of 1% trifluoroacetic acid (TFA). Unless and otherwise mentioned, LcA concentration and the time of incubation were adjusted so that no more than 20% of the substrate was converted into products.

The amounts of uncleaved 17-mer substrate and the products were measured after separation using a Waters Acquity UPLC™ system employing a reverse-phase C18 column (2.1×50 mm, 1.7-µm particle size) with 0.1% TFA in H_2_O as solvent A and 70% acetonitrile-0.1% TFA as solvent B. The peptides were eluted at a flow rate of 0.5 ml/min with a linear gradient of 10%B to 42%B over one min , 0.5 min after injection of 5 µl sample [27]. Column regeneration was for 0.7 min [27]. The 17-mer substrate, its N-terminal product, and its C-terminal product were eluted at 1.75, 1.3, and 0.9 min, respectively, and were completely separated [27]. When analyzed in the Waters HPLC column (see later), the reaction mixtures yielded identical results. However because of rapid analysis of minute sample volumes using little solvent, UPLC™ [27] was used in analyzing the 17-mer substrate reactions. The UPLC™ system was not suitable to resolve the products from the full length and the 66-mer substrates. Therefore, these reaction mixtures were analyzed on a Waters C18 (Synergy) 4.6×75 mm (3–5 µm particle size) HPLC column with a linear gradient of 10%B to 90%B over 12.5 min, one minute after injection of 100 µl sample, followed by a jump to 100%B that was held for 3 min. The smaller C-terminal products from these substrates eluted at 5.6 min while the larger N-terminal product and the substrates eluting between 11–12 min were not completely separated. Therefore, we used the area under the C-terminal products for accurate quantification of the reaction.

The UPLC™ was equipped with Waters Acquity™ Sample Manager (autosampler), a Waters Acquity™ photodiode array detector and Empower Pro software, while the Waters HPLC was equipped with Waters 717plus autosampler, a Waters 996 photodiode array detector, and Empower Pro software. Quantification of peptides was based on the area under their 210 nm absorbance peaks. The limit of detection in 5 µl UPLC™-injected sample for the 17-mer substrate and its products was 5 µM [27]. The limit of detection in 100 µl HPLC-injected sample of the C-terminal product from SNAP25 and its 66-mer substrates was 0.1 µM.

Insolubility of the substrates limited our ability to use saturating substrate concentration in the kinetic experiments. Therefore we used several concentrations of each substrate around their previously reported *K_m_* values. Kinetic parameter calculations used a “one substrate, two product” model (E + S ↔ ES → E + P1 +P2) ignoring the on and off rates of the substrates and products since they were not determined. *K_m_* and *k_cat_* were determined by hyperbolic curve-fitting of the experimental data at various substrate concentrations using the Michaelis-Menten equation, *k_obs_ = *(*k_cat_*×[S])/(*K_m_*+[S]), in a KaleidaGraph (Synergy Software, Reading, PA) software package.
